# Nanostructured Lipid Carriers Deliver Resveratrol, Restoring Attenuated Dilation in Small Coronary Arteries, via the AMPK Pathway

**DOI:** 10.3390/biomedicines9121852

**Published:** 2021-12-07

**Authors:** Cai Astley, Chahinez Houacine, Azziza Zaabalawi, Fiona Wilkinson, Adam P. Lightfoot, Yvonne Alexander, Debra Whitehead, Kamalinder K. Singh, May Azzawi

**Affiliations:** 1 Centre for Bioscience, Department of Life Sciences, Manchester Metropolitan University, Chester Street, Manchester M1 5GD, UK; caiastley.ca@protonmail.com (C.A.); azziza.z.zaabalawi@stu.mmu.ac.uk (A.Z.); f.wilkinson@mmu.ac.uk (F.W.); A.Lightfoot@mmu.ac.uk (A.P.L.); Y.Alexander@mmu.ac.uk (Y.A.); 2 School of Pharmacy and Biomedical Sciences, Faculty of Clinical and Biomedical Sciences, University of Central Lancashire, Preston PR1 2HE, UK; chahinez.houacine1@gmail.com; 3 Department of Natural Sciences, Manchester Metropolitan University, Chester Street, Manchester M1 5GD, UK; D.Whitehead@mmu.ac.uk

**Keywords:** resveratrol, endothelium, coronary artery, oxidative stress, reactive oxygen species, nanostructured lipid carriers

## Abstract

Nanostructured lipid carriers (NLCs) are an emerging drug delivery platform for improved drug stability and the bioavailability of antihypertensive drugs and vasoprotective nutraceutical compounds, such as resveratrol (RV). The objective of this study was to ascertain NLCs’ potential to deliver RV and restore attenuated dilator function, using an ex vivo model of acute hypertension. Trimyristin–triolein NLCs were synthesized and loaded with RV. The uptake of RV-NLCs by human coronary artery endothelial cells (HCAECs) maintained their viability and reduced both mitochondrial and cytosolic superoxide levels. Acute pressure elevation in isolated coronary arteries significantly attenuated endothelial-dependent dilator responses, which were reversed following incubation in RV-NLCs, superoxide dismutase or apocynin (*p* < 0.0001). RV-NLCs demonstrated a five-fold increase in potency in comparison to RV solution. At elevated pressure, in the presence of RV-NLCs, incubation with Nω-nitro-l-arginine (L-NNA) or indomethacin resulted in a significant reduction in the restored dilator component (*p* < 0.0001), whereas apamin and TRAM-34 had no overall effect. Incubation with the adenosine monophosphate-activated protein kinase (AMPK) inhibitor dorsomorphin significantly attenuated dilator responses (*p* < 0.001), whereas the SIRT-1 inhibitor EX-527 had no effect. RV-NLCs improved the impaired endothelial-dependent dilation of small coronary arteries, following acute pressure elevation, via NO and downstream COX elements, mediated by AMPK. We suggest that RV-NLCs are an effective delivery modality for improved potency and sustained drug release into the vasculature. Our findings have important implications for the future design and implementation of antihypertensive treatment strategies.

## 1. Introduction

Nanostructured lipid carriers (NLCs) are an emerging drug delivery platform for the treatment of cardiovascular diseases, including hypertension. NLCs have been shown to increase the bioavailability of poorly soluble antihypertensive drugs [1,2] and vasoprotective nutraceutical products, such as resveratrol (RV) [3,4], protecting it from rapid metabolism, whilst also allowing fine manipulation of release kinetics [5,6,7,8]. RV (a polyphenolic phytoalexin compound) has been shown to enhance blood pressure reduction when combined with traditional antihypertensive therapy. This is attributed to its antioxidant and anti-inflammatory properties [8], including scavenging a range of reactive oxygen species (ROS) moieties [9]; upregulating the expression of several antioxidant enzymes [10]; and increasing nitric oxide (NO) production [11] via activation of SIRT1 (silent mating type information regulation 2 homolog) [12,13]. The involvement of AMPK (adenosine monophosphate-activated protein kinase) as an alternate target has also been proposed [14]. We have also recently demonstrated that RV can potentiate endothelial-dependent dilator responses in aortic vessels and femoral arteries from young and aged mice [15]. In human studies, we and others have shown that RV improves flow-mediated dilation (FMD) in cardiovascular disease patients after coronary artery bypass graft surgery [16]; improves ventricular systolic and diastolic function; and decreases cholesterol levels after myocardial infarction. However, other studies have reported inconclusive or contradictory findings, which may be due to insufficient RV bioavailability in vivo, particularly following oral administration, with <1% available globally [17,18,19,20]. Despite RV’s good absorption profile (75% uptake), the resulting low plasma concentration leads to inadequate systemic distribution and limited exposure to specific activation sites, resulting in insufficient pharmacological effects. This lack of bioavailability is widely considered to be a result of RV’s metabolic instability, resulting from multiple rapid glucuronidation and sulfation reactions during phase I and II metabolism [21]. As a consequence of its low bioavailability and poor stability [15], it is evident that alternative modes of RV administration are required.

Using isolated vessels ex vivo, we have previously demonstrated that nanoparticles composed of mesoporous silica are rapidly taken up by the lining endothelial cells and can be used to improve vasodilator potential [22,23]. Though mesoporous silica is a promising drug carrier, it suffers from limitations, including low colloidal stability and aggregation in physiological solutions, reducing circulation time and preventing desirable cell uptake. NLCs, the latest generation of lipid nanoparticle systems, are composed of mixtures of liquid and solid lipids. They have distinct advantages over inorganic nanoparticles and their previous generation counterparts (such as liposomes and solid lipid NPs), including improved drug stability, biocompatibility, tissue uptake and sustained drug delivery [24]. The present research utilized solid lipid trimyristin-based NLCs stabilized with C18:1 triglyceride, triolein as a liquid lipid. The three bent carbon chains of triolein disrupt the packing of trimyristin crystals offering better stability and higher drug loading [25]. Further, triolein, a triacylglycerol, can modulate cell membranes for better uptake [26]. The objective of this study was to ascertain the potential of trimyristin–triolein NLCs as a modality to deliver RV (as a model drug) into the vasculature, using an ex vivo model of acute hypertension. We hypothesize that RV, delivered via NLCs, enables the restoration of attenuated dilation after acute pressure elevation ex vivo and provides a more sustained dilator response than RV solution alone.

## 2. Materials and Methods

### 2.1. Reagents and Chemicals

Resveratrol (RV) was obtained from Manchester organics, UK. For NLC synthesis, trimyristin (Dynasan 114) was kindly donated as a gift sample from Cremer oleo division, UK. Triolein, sodium cholate, Tween-80, Span-80 and coumarin-6 were purchased from Sigma-Aldrich, UK. Phosphatidylcholines were obtained from Lipoid oleo division, UK. All reagents were of analytical grade. Deuterated DMSO was obtained from GOSS Scientific, UK. Physiological salt solution (PSS) (composition (mM): 119 NaCl, 4.7 KCl, 1.2 MgSO_4_7H_2_O, de-ionized (dH_2_O), 25 NAHCO_3_, 1.17 KHPO_4_, 0.03 K_2_EDTA, 5.5 glucose, 1.6 CaCl_2_H_2_O; pH 7.4) [22,23].

### 2.2. NLC Synthesis and Characterization

Blank (non-loaded), RV and RV–dye-loaded trimyristin–triolein NLCs were prepared using a hot melt emulsification technique [27]. The water phase containing surfactants, sodium cholate (0.25%) and Tween-80 (1%) was heated to 70 °C prior to amalgamation with the melted lipid phase comprising trimyristin (1.5%), triolein (0.5%), phosphatidylcholines (0.5%), Span-80 and RV (1 mg/mL). The dispersion was mixed under magnetic stirring for 5 min. The pre-emulsion was sonicated using a probe sonicator (Fisherbrand™ Q700) for 15 min. The resulting emulsion was allowed to cool down at ambient temperature overnight to form the NLCs. For the dye-loaded RV-NLCs, coumarin-6 dye was dissolved in the lipid phase with RV, allowing entrapment within the NLCs. The RV-NLC dispersion was centrifuged at 1200× *g* for 5 min at 25 °C in order to remove the residual titanium produced during probe sonication.

Particle size and polydispersity index (PDI) of RV-NLCs were measured using a diffraction light scattering technique (DLS) on Malvern Zetasizer (Nano ZS, Malvern Ltd., Worcestershire, UK) at a scattering angle of 173°. Absorption spectra for RV-NLCs were acquired by a UV–Vis spectrophotometer (Jenway 7305, Cole-Palmer, Vernon Hills, IL, USA), and fluorescence spectra were recorded by a fluorescence spectrometer (F-7000, Hitachi, Tokyo, Japan).

The amount of free drug was determined by an ultrafiltration method with a 3 KDa molecular weight cut-off. Briefly, RV-NLC dispersion was placed into a centrifugal filter tube and centrifuged at 13,000 rpm for 60 min. After centrifugation, the amount of soluble free drug in the aqueous phase was quantified by a high-performance liquid chromatography (HPLC) method using a C18 Luna column with detection at 306 nm. The total drug in the RV-NLCs was determined by adding 4 mL of tetrahydrofuran to 1 mL of nanoparticle dispersion to ensure that the lipid and the drug were completely dissolved. The solution was filtered through a 0.22 nm filter diluted with the mobile phase and analyzed by HPLC. Entrapment efficiency (EE) and drug loading (DL) were calculated as follows:

(1) $$\mathrm{EE}\left(\%\right)=\frac{W_{\mathrm{total}}-W_{\mathrm{free}}}{W_{\mathrm{total}}}\times 100\%$$

(2) $$\mathrm{DL}\left(\%\right)=\frac{W_{\mathrm{total}}-W_{\mathrm{free}}}{W_{\mathrm{Lipids}}}\times 100\%$$

where W_free_ is the amount of free drug in the supernatant; W_total_ is the amount of RV added; W_lipids_ is the total amount of lipids in the formulation.

Furthermore, RV-NLCs were characterized by Fourier transform infrared spectroscopy (FTIR), XRD and NMR. Infrared spectra of samples (RV alone, lyophilized blank NLCs, lyophilized RV-NLCs) were scanned on an FTIR (Nicolet 380 FTIR Spectrometer, Thermo Scientific, Waltham, MA, USA) in a frequency range between 4000 and 400 cm^−1^ using the KBr pellet method [28]. OMNIC 8.0.380 computerized FTIR Spectroscopy software (ThermoScientific) was used to acquire and analyze the spectrum. 1H-NMR spectra were obtained on an NMR Bruker Fourier (Germany), operating at 300 MHz and 20 °C. Tetramethyl silane (TMS) was used as a reference for 0 ppm NMR. NMR spectra were processed employing MestReNova (10.0.2 software, Spain). XRD was conducted by loading the samples (RV, trimyristin, lyophilized RV-NLCs and cryoprotectant trehalose) onto inert polymeric discs and scanning them from 5 to 50° with a set scan type, coupled with two θ (theta) on an X-ray diffractor, D2 PHASER, Bruker, Germany, using a scintillation counter and a one-dimensional LYNXEYE detector.

RV-NLC stability was assessed in physiological salt solution (PSS), in which ex vivo coronary artery experiments were conducted. RV-NLCs were incubated in PSS, and samples were withdrawn at predetermined time points and assessed for particle size, polydispersity index and zeta potential using a Zetasizer Nano ZS system (Malvern Instruments, Malvern, UK) [27]. To determine the release rate of RV from NLCs, a dialysis tubing diffusion method was employed. Dialysis bags (Spectra/Por^®^ 3 Dialysis Tubing, molecular weight cut-off 3.5 kD) were washed with distilled water and saturated overnight in release medium. Then, 5 mL of RV-NLC dispersion was added to the dialysis bag and placed in a glass vessel (ERWEKA^®^ dissolution tester, D-63150 Heusenstamm/Germany) containing 900 mL of dissolution media (pH7.4) at 37 ± 0.5 °C. Samples of 1 mL were withdrawn at predetermined time intervals, diluted with mobile phase (acetonitrile: water) and analyzed using HPLC on a C18 Luna column with detection at 306 nm [29].

### 2.3. Cell Culture Studies 

Human coronary artery endothelial cells (HCAECs) were obtained from PromoCell (Heidelberg, Germany). Cells were grown in endothelial cell growth medium (MV2) supplemented with an MV2 supplement pack, 100 µL/mL penicillin and 100 µL/mL streptomycin. Cultures were maintained at 37 °C with 5% CO_2_ in a humidified incubator. Cells were used at passages 3–6. The viability of HCAECs was assessed using the fluorometric assay, Alamar blue, according to the manufacturer’s instructions (ThermoFisher, Schiphol, UK). Briefly, cells were cultured in 96-well plates at a density of 1 × 10^4^ cells/well for 24 h. Cells were exposed to cumulative concentrations of blank, RV-loaded and RV–dye-loaded NLCs and RV solution (90 µM, 45 µM, 4.5 µM, 0.45 µM, 0.045 µM) for 24 h. Negative controls were incubated with 0.1% Triton-X100 (Sigma, UK). Alamar blue was added at a 1:10 ratio. HCAECs were incubated in the dark at 37 °C for 4 h. Fluorescence was measured at ex. 570/em. 590 using a BioTek SynergyTM HT microplate reader. Cell viability was calculated by normalizing treatment samples against positive controls (untreated cells).

To determine NLC uptake, cells were cultured on glass slides mounted with ProPlate^®^ multi-well chambers at a density of 1 x 10^4^/well. Cells were incubated in media supplemented with 0.45 µM dye-labeled RV-NLCs and left for 30 min and then fixed with 4% paraformaldehyde (PFA). Cells were washed in PBS-Tween (0.05%) to permeabilize cells. Phalloidin (1:10,000) was used to stain actin filaments, and Vectashield^®^ antifade mounting medium containing DAPI as a nuclear counterstain was used and sealed using a coverslip (Vector Laboratories, UK). Slides were imaged using a Zeiss fluorescent microscope under ×20 magnification.

The generation of mitochondrial and cytosolic superoxide was quantified using MitoSOX™ Red mitochondrial superoxide indicator and dihydroethidium (DHE), respectively (Thermo, UK). Briefly, HCAECs were cultured in 96-well plates at a density of 1 × 10^4^ cells/well for 24 h. Based upon HCAEC tolerances previously assessed following exposure to hydrogen peroxide (H_2_O_2_; Supplementary Figure S1), cells were exposed to 100 µM of H_2_O_2_ in media to stimulate ROS generation for 30 min [30]. Cells were washed with phosphate-buffered saline, followed by fresh medium in the presence/absence of RV-NLCs for 30 min (0.45 µM). Following preparation and treatment, cells were incubated with MitoSOX (5 µM) or DHE (10 µM) for 15 min. Fluorescence was measured at ex. 530/em. 590 using a BioTek SynergyTM HT microplate reader. Fluorescence values were normalized to control wells (untreated).

### 2.4. Vascular Function Studies

First-order septal coronary arteries (100–200 µm) were excised from male Wistar rats (150–250 g; 8 weeks old), euthanized by stunning and cervical dislocation in accordance with the ‘Animals (Scientific Procedures) Act 1986′ and institutional guidelines. Arteries were mounted between two glass cannulas on a pressure myograph chamber (Living Systems, St Albans City, VT, USA) and pressurized to an intravascular pressure of 60 mmHg using a servo control unit (Living Systems Instrumentation, St Albans City, VT, USA), as previously described [15]. All arteries were pre-constricted in serotonin sub-maximally (5-HT, 10^−6^ M; Supplementary Figure S2). Endothelial-dependent acetylcholine (ACh; 10^−9^–10^−3^ M) responses were assessed prior to and following acute pressure elevation. Vessels were exposed to elevated pressure (150 mmHg) for 30 min, and then returned down to 60 mmHg for a further 30 min, in the presence/absence of RV-NLCs (0.45 µM) or RV solution (0.45 µM).

In a separate set of experiments, the sustained response, 1 h after the pressure elevation, was also assessed. To evaluate the possible contribution of ROS to the attenuation of endothelial-dependent dilation, vessels were co-incubated with superoxide dismutase (SOD, 300 U^-^mL) and apocynin (NADPH oxidase inhibitor; 30 µM). Endothelial-independent responses (sodium nitroprusside; SNP, 100 µM; papaverine; PAPA, 100 µM) were assessed at the end of each experiment. Inhibition studies were used to determine the influence of RV-NLCs on the vasodilator pathways. Responses were assessed in a separate set of experiments, following incubation in the nitric oxide synthase inhibitor Nω-nitro-L-arginine (L-NNA, 100 µM) and the small (SK) and intermediate (SK) calcium-activated potassium channel blockers apamin (100 µM) and TRAM-34 (1 µM), as well as the cyclooxygenase (COX) inhibitor, indomethacin. The effect of inhibitors on vessel function was assessed alone, in combination or in the presence/absence of RV-NLCs. Responses were assessed in the continued presence of inhibitors in the superfusate. The mechanistic contributions of AMPK and SIRT-1 were assessed following incubation with the inhibitors dorsomorphin and EX-527, respectively. To assess whether RV-NLCs act via endothelial-independent mechanisms, the endothelium was denuded prior to beginning the experiments.

### 2.5. Statistical Analysis

All statistical analyses were performed using Prism version 8.10 for Windows, GraphPad Software, La Jolla, CA, USA. DLS measurements were analyzed using a one-way analysis of variance (ANOVA) followed by Tukey multiple comparisons. Drug release was analyzed by a two-way ANOVA followed by Sidak multiple comparisons. Cell viability in response to H_2_O_2_ and RV-NLCs was analyzed by a one- and two-way ANOVA, respectively, followed by a Tukey multiple comparison. Superoxide generation was analyzed by a two-way ANOVA followed by a Sidak multiple comparisons. Dilator responses elicited by ACh in the presence of RV or PSS ± treatment were analyzed using a two-way ANOVA followed by Sidak multiple comparisons. To calculate EC_50_ values, data were normalized, transformed and plotted using a nonlinear regression model. LogEC_50_ values were calculated and compared using an extra sum-of-squares F test. Values of *p* < 0.05 were considered significant. Data are represented as means ± standard error of mean (SEM) unless stated otherwise.

## 3. Results

### 3.1. Sustained-Release RV-NLCs Are Stable in Physiological Solution

**Figure 1:**
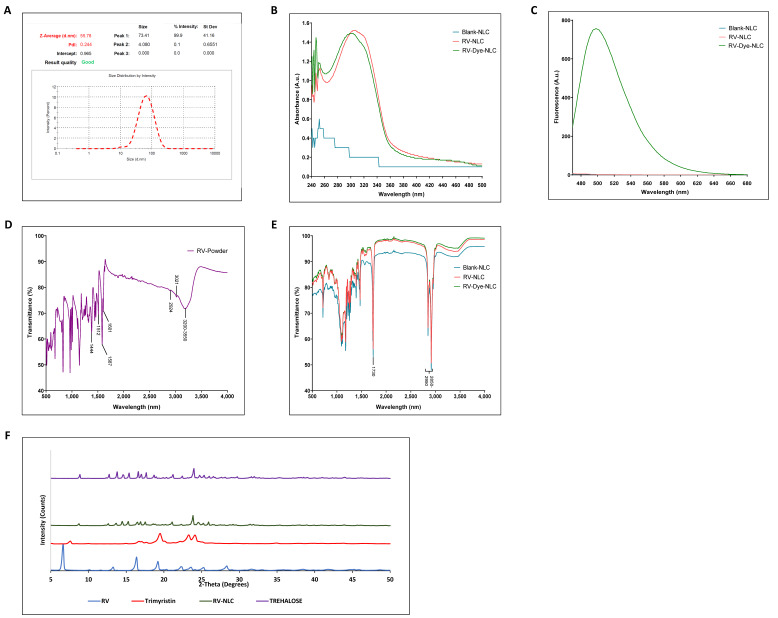
Characterization of RV–dye-encapsulated NLCs. (**A**) Particle size determination by dynamic light scattering (DLS). (**B**) Confirmation of resveratrol (RV) entrapment using ultraviolet–visible spectroscopy (UV–Vis). The presence of resveratrol (RV) within RV-NLCs was confirmed by the presence of a characteristic absorbance peak at 300 nm, which was absent from blank NLCs. (**C**) Confirmation of dye entrapment. The presence of dye (coumarin-6) was confirmed using fluorescence spectroscopy, with a characteristic fluorescence peak at 500 nm, which was absent from blank and RV-NLCs. (**D**,**E**) Confirmation of entrapment and molecular structure. Fourier transform infrared spectroscopy (FTIR) spectra of RV-powder and NLCs, respectively, displaying transmittance (%) between 500 and 4000 nm. Characteristic absorptions are indicated on figures (for interpretation of the references, refer to main text). (**F**) X-ray powder diffractogram shows the amorphous nature of embedded RV in the NLCs with absence of RV peaks in the RV-NLCs.

RV-loaded NLCs were synthesized by the simple and scalable technique of melt emulsification. The DLS revealed the formation of uniform NLCs with a mean hydrodynamic diameter of 55.78 nm with a PDI of 0.244 (Figure 1A) and zeta potential of −25.6, indicating a product with good stability. Ultraviolet–visible spectroscopy demonstrated a characteristic absorbance spectrum of RV (*trans*-resveratrol) at 304 nm [31]. RV and RV–dye-loaded NLCs both had spectral peaks at approximately 304 nm, which was characteristically absent from the blank NLCs (Figure 1B). Fluorescence peaks were detected at 500 nm in RV–dye-loaded NLCs, confirming coumarin-6 loading (Figure 1C). The RV-NLCs showed a high EE of 97.058 ± 0.057% with a drug loading of 3.4%. FTIR provides a powerful means of material characterization. The strong, broad-band characteristic for trans-resveratrol can be observed between 3550 and 3200 cm^−1^, originating from the valence ν(OH)vibrations of phenolic groups. The band at 3021 cm^−1^ is the result of the valence vibration of ethenyl groups (=C-H), while the bands at 2924 and 2852 cm^−1^ originate from the valence vibrations of C-H bonds from CH and CH_2_ groups [32]. Phenolic compounds are consistently accompanied by characteristic aromatic hydrocarbons (benzene). The valence vibrations ν(C=C) of the benzene ring were observable at 1606, 1587, 1512 and 1444 cm^−1^ (Figure 1D). The infrared spectrum of RV-NLCs highlights peaks corresponding to its parent lipid constituents, with peaks at 2950–2850 and 1730 cm^−1^ corresponding with the presence of alkane (C-H) and carbonyl ester groups (C=O), consistent with the lipid components trimyristin and triolein, respectively (Figure 1E). However, the peaks of RV were absent in the FTIR of the RV-NLCs, presumably because of molecular dispersion or the complete entrapment of RV within the lipid matrix of NLCs [33]. This was further confirmed by the diffraction pattern of RV-NLCs, which showed the absence of RV peaks at 2Ɵ values of 6.575°, 13.178°, 16.308°,19.213°, 22.386°, 23.659°, 25.274° and 28.263°. The RV-NLCs diffraction pattern showed peaks at 2Ɵ values of 8.857°, 12.774° 13.804°, 14.571°, 15.399°, 16.631°, 16.995°, 17.641°, 18.630°, 21.195°, 22.407° and 23.941° and demonstrated the presence of trehalose used as a cryoprotectant in NLCs; as a result, the peaks of trimyristin (7.524°,16.611°, 19.398°, 23.174° and 24.062°) were overshadowed by the high concentration of trehalose (Figure 1F).

**Figure 2:**
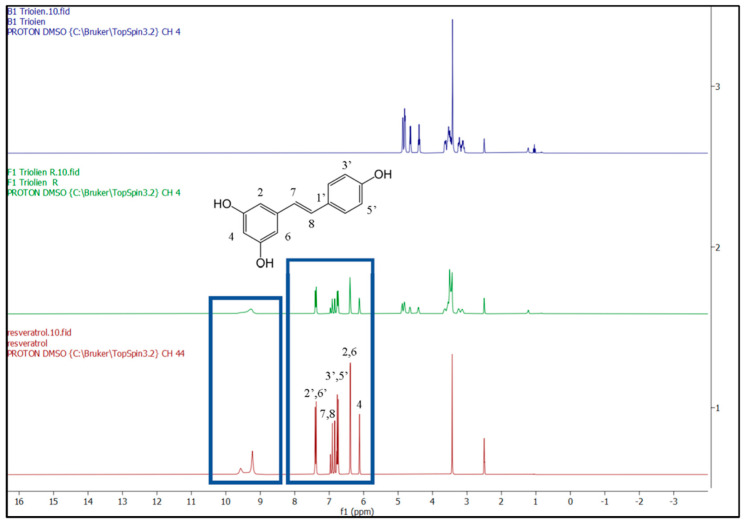
1H-NMR spectra (500 MHz) of resveratrol (red pattern), RV-NLCs (green pattern) and blank NLCs (blue pattern) in DMSO. Carbon-bound protons appeared in the region between 6.1 and 7.07 ppm, while the OH protons appeared in the region between 9 and 9.5 ppm in the 1H-NMR spectrum of the RV-NLCs, indicating the presence of the drug in the RV-NLC formulations. Drug peaks in NLCs were identical to the peaks of the drug in the bulk. No chemical shift was observed when the drug was encapsulated in the NLC, indicating that the drug was present in an intact form. No degradation of the drug was evident upon its formulation into the NLCs.

NMR is one of the main spectroscopic techniques employed in the characterization of the molecular structure of different chemical entities and detailed conformational dynamics at the atomic level. 1H-NMR spectra were obtained from RV, blank and RV-NLCs dissolved in deuterated DMSO (Figure 2). The carbon-bound protons appeared in the region between 6.1 and 7.07 ppm, while the OH protons appeared in the region between 9 and 9.5 ppm in the 1H-NMR spectrum of the RV-NLCs, indicating the presence of the drug in the RV-NLC formulations. In addition, the drug peaks in NLCs were identical to the peaks of the drug in the bulk. No chemical shift was observed when the drug was encapsulated in the NLC, indicating that the drug was present in an intact form. No degradation of the drug was evident upon its formulation into the NLCs [34].

The dilution of RV-NLCs (0.45 µM) in PSS led to an increase in their hydrodynamic diameter (96.64 nm; *p* < 0.0001; Supplementary Figure S3A). This increase in size can be attributed to the attractive interactions between the nanoparticle surface and solvent molecules/ions present in the PSS resulting in an ionic liquid-based supramolecularly structured shell that surrounds the nanoparticles. The presence of surface ions on the NLCs was further confirmed by the drop in zeta potential (−8.96) when incubated with PSS. These observations are in agreement with those of previous reports [35,36,37]. However, once the ionic layer was established, the NLCs remained stable with no aggregation or time-dependent increase in particle size (Supplementary Figure S3B) [35]. RV release from NLCs was sustained, with the maximal release of RV observed after 12 h (27.7%) compared to the diffusion of the RV solution (maximum diffusion 73.71% at 11 h). This confirms the ability of NLCs to retain the entrapped drug within the NLC matrix and protect it, reducing its release and degradation in the outer media (Supplementary Figure S3C) [36].

### 3.2. RV-NLCs Maintain Endothelial Cell Viability and Diminish H_2_O_2_ -Induced Superoxide Generation In Vitro

**Figure 3:**
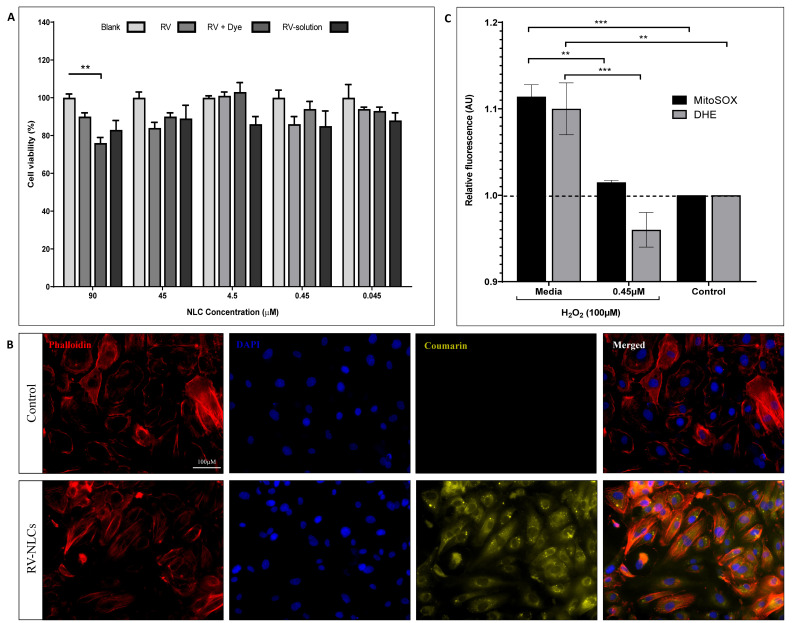
(**A**) Effect of RV-NLCs on cell viability. Human coronary artery endothelial cell (HCAEC) viability following exposure to NLCs (blank, RV-loaded and RV–dye-loaded NLCs) at concentrations ranging between 90 and 0.045 µM over 24 h. Cell viability determined using an Alamar blue assay (*n* = 3). Data normalized to control wells (untreated). NLCs had no significant effect on cell viability, other than at 90 µM (*p* < 0.01). (**B**) RV-NLC uptake within HCAECs. Representative fluorescence micrographs of HCAECs. Cultured cells were incubated with growth medium or RV–dye-loaded NLCs for 30 min, followed by DAPI (nuclear) and phalloidin (actin) staining. Magnification x20. Images acquired from 5 representative slides. (**C**) Effect of RV-NLCs on HCAEC mitochondrial (MitoSOX) and cytosolic (dihydroethidium; DHE) superoxide. MitoSOX and DHE fluorescence quantification highlighting superoxide generation in HCAECs following 30 min exposure to H_2_O_2_ (100 µM). Cells were co-incubated with RV-NLCs (0.45 µM) or media alone. Data expressed as percentage change from control (untreated cells—no H_2_O_2_/RV; *n* = 3). H_2_O_2_ exposure significantly elevated mitochondrial and cytosolic superoxide levels in HCAECs (*p* < 0.001; *p* < 0.01) and was reduced following incubation with RV-NLCs (*p* < 0.01; *p* < 0.0001). Cell viability and superoxide generation were analyzed by a two-way ANOVA, followed by a Tukey and Sidak multiple comparisons, respectively. Data are presented as mean ± SEM. ** *p* < 0.01. *** *p* < 0.001.

Following 24 h co-incubation of RV-NLCs or RV solution with HCAECs, no significant change in cell viability was observed (Figure 3A); however, at the highest dose (90 µM), a significant reduction in viability of 23% was detected between blank and RV–dye-loaded NLCs (*p* < 0.01). Fluorescence microscopy demonstrated that RV–dye-loaded NLCs (0.45 µM) were taken up by >90% of HCAECs after 30 min (Figure 3B). H_2_O_2_ exposure led to a significant increase in mitochondrial and cytosolic superoxide generation in the endothelial cells compared to controls (*p* < 0.001; *p* < 0.01, respectively). A significant reduction in mitochondrial and cytosolic superoxide generation was observed in cells incubated with 0.45 µM RV-NLCs in comparison to those treated with media alone (*p* < 0.01; *p* < 0.0001, respectively). Fluorescence values were normalized to control wells (untreated) (Figure 3C). In vitro studies demonstrated that NLCs are well tolerated across a range of concentrations, whilst also displaying good uptake and antioxidant capability following 30 min incubation with 0.45 µM RV-NLCs. To mitigate potential future pharma toxicological effects, *ex vivo* studies were conducted using this concentration.

### 3.3. Acute Pressure Elevation Attenuates Endothelial-Dependent Dilator Responses via the Activation of NADPH Oxidase

**Figure 4:**
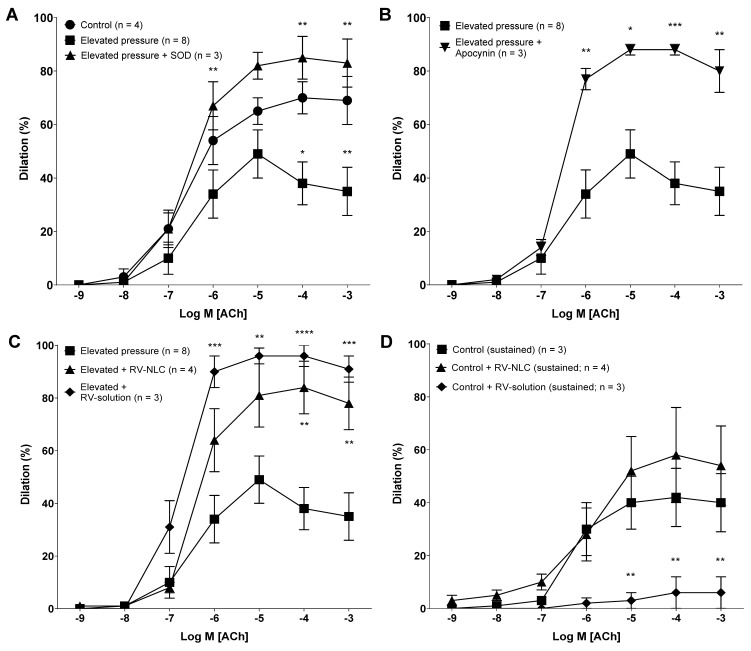
Endothelial-dependent acetylcholine (ACh) induced dilator responses in the coronary arteries of young Wistar rats (2 months old). (**A**) Effects of elevated pressure ± superoxide dismutase (SOD) on ACh-induced dilation. Elevated pressure significantly attenuated dilator responses (*p* < 0.01), which were restored following co-incubation with SOD (*p* < 0.0001). Control (*n* = 4); elevated pressure (*n* = 8); elevated pressure + SOD (*n* = 3). (**B**) The influence of the NADPH oxidase inhibitor apocynin (30 µM) on ACh-induced dilation following acute pressure elevation. Incubation with apocynin resulted in restoration of dilation (*p* < 0.0001). Elevated pressure (*n* = 8); elevated pressure + apocynin (*n* = 3). (**C**,**D**) The influence of resveratrol–nanostructured lipid carriers (RV-NLCs)/RV solution on initial and sustained ACh-induced dilation following acute pressure elevation. RV-NLCs and RV solution significantly improved attenuated dilation (*p* < 0.0001 and *p* < 0.0001, respectively). Sustained responses of RV-NLCs were maintained at levels comparable to those of control tissue in comparison to RV solution (*p* < 0.0001). Two-way ANOVA followed by Sidak multiple comparisons test. Data are presented as mean ± SEM.* *p* < 0.05. ** *p* < 0.01. *** *p* < 0.001. **** *p* < 0.0001.

Initial studies were conducted to demonstrate that acute pressure elevation attenuates the magnitude of dilator response in isolated coronary arteries. All vessels constricted to high potassium and serotonin solution. All serotonin pre-constricted (sub-maximal; 5-HT 10^−6^ M) vessels dilated to ACh in a dose-dependent manner. Following elevated pressure (150 mmHg), a significant reduction in ACh responses was observed (*p* < 0.01), likely attributable to a rise in ROS, which was confirmed by the restoration of endothelial-dependent dilation after co-incubation with SOD (from bovine erythrocytes) (*p* < 0.0001; Figure 4A) or apocynin (*p* < 0.0001; Figure 4B).

### 3.4. RV-NLCs Restore the Magnitude of Dilation Following Acute Pressure Elevation

In the next set of experiments, the influence of RV-NLCs on the magnitude of dilator responses following acute pressure elevation was examined. Acute pressure elevation followed by incubation with 0.45 µM RV-NLCs resulted in a significant improvement in endothelium-dependent (ACh) dilation in comparison to acute pressure elevation alone (*p* < 0.0001). Similar responses were observed following incubation with 0.45 µM RV solution (*p* < 0.0001; Figure 4C). A significant shift in LogEC_50_ values was observed between the initial RV-NLC and RV solution responses, with mean values of 4.73 × 10^−7^ M (0.47 µM) and 1.16 × 10^−7^ M (0.16 µM), respectively (*p* < 0.0001; Supplementary Figure S4A). Sustained responses were reduced for both RV-NLCs and RV solution one-hour post-incubation when compared to initial responses (Figure 4D). However, endothelium-dependent responses were maintained at levels comparable to those of control tissue with RV-NLCs in comparison to RV solution (*p* < 0.0001). A significant rightward shift in LogEC_50_ values was observed, with RV-NLCs showing increased potency in comparison to RV solution, with values of 1.13 × 10^−6^ M (1.13 µM) and 5.33 × 10^−6^ M (5.33 µM), respectively (*p* < 0.01; Supplementary Figure S4B).

### 3.5. RV-NLCs Restore Dilation via NO and COX following Elevated Pressure, Mediated via AMPK

**Figure 5:**
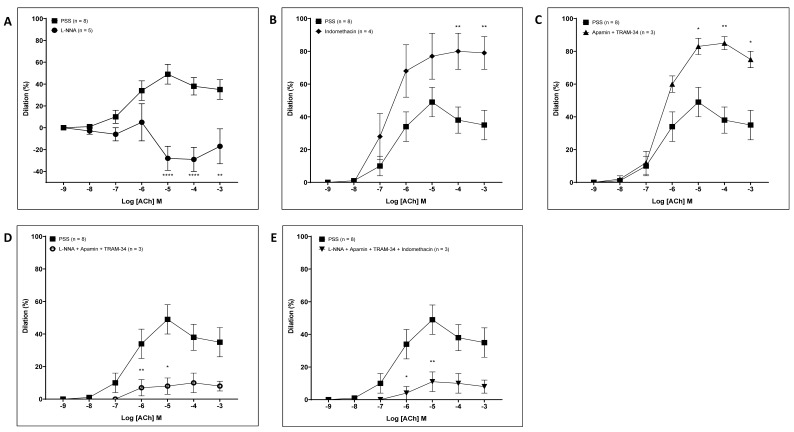
Characterization of the dilator response following acute pressure elevation. The influence of the inhibitor cocktails of NΩ-nitro-L-arginine (**A**; 100 µM; *n* = 3); indomethacin (**B**; 10 µM; *n* = 4); apamin (100 nM) and TRAM-34 (1 µM) (**C**; *n* = 3); L-NNA, apamin and TRAM-34 (**D**; *n* = 3); and L-NNA, indomethacin, apamin and TRAM-34 in combination (**E**; *n* = 3). Incubation with L-NNA resulted in a significant constriction (*p* < 0.0001). Incubation with the cyclooxygenase (COX) 1 and 2 inhibitor, indomethacin, resulted in a significant potentiation of dilation (*p* < 0.0001). Incubation with apamin and TRAM-34 resulted in the potentiation of dilation (*p* < 0.0001). Co-incubation of vessels with L-NNA and EDHF inhibitors (apamin and TRAM-34) resulted in attenuated dilation (*p* < 0.0001). Incubation with L-NNA, apamin, TRAM-34 and indomethacin resulted in the loss of dilator component (*p* < 0.0001). Two-way ANOVA followed by Sidak multiple comparisons test. Data are presented as mean ± SEM.* *p* < 0.05. ** *p* < 0.01.

To characterize the endothelium-dependent dilator component of coronary arteries, following acute pressure elevation, we initially carried out inhibition studies in the absence of RV-NLCs. Incubation with L-NNA, a nitric oxide synthase inhibitor, resulted in a significant constriction (*p* < 0.0001; Figure 5A), suggesting that a basal release of NO has an important dilator influence on the coronary vasculature [38]. Following incubation with the nonselective inhibitor of cyclooxygenase (COX) 1 and 2, indomethacin, a significant potentiation of dilation is observed (*p* < 0.0001; Figure 5B). Incubation with the small and intermediate calcium-activated potassium channel blockers apamin and TRAM-34 resulted in the potentiation of dilation (*p* < 0.0001; Figure 5C). The co-incubation of vessels with L-NNA and EDHF inhibitors (apamin and TRAM-34) resulted in attenuation, suggesting that the potentiation observed following incubation with EDH inhibitors may be a compensatory mechanism by L-NNA (*p* < 0.0001; Figure 5D). Incubation with L-NNA, apamin, TRAM-34 and indomethacin resulted in the loss of the dilator component (*p* < 0.0001; Figure 5E).

**Figure 6:**
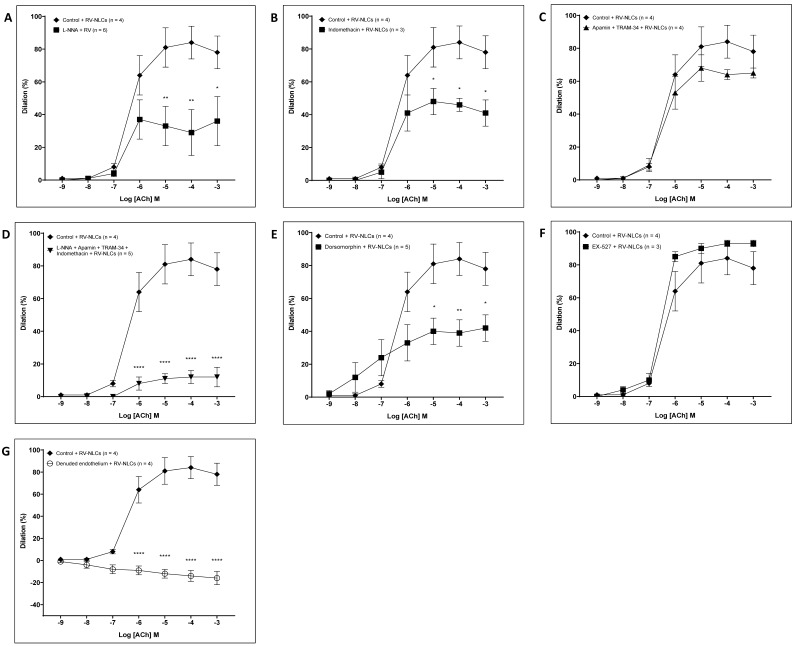
Characterization of the dilator response following acute pressure elevation in the presence of resveratrol–nanostructured lipid carriers (RV-NLCs; 0.45 µM). The influence of the inhibitor cocktails on the acetylcholine-induced relaxation in serotonin pre-constricted vessels: NΩ-nitro-L-arginine (**A**; 100 µM; *n* = 6); indomethacin (**B**; 10 µM; *n* = 3); apamin (100 nM) and TRAM-34 (**C**; 1 µM; *n* = 4); L-NNA, indomethacin, apamin and TRAM-34 (**D**; *n* = 5); dorsomorphin (**E**; 20 µM; *n* = 5); and EX-527 (**F**; 10 µM; *n* = 3). Incubation with L-NNA resulted in significant constriction (*p* < 0.0001). Incubation with indomethacin resulted in significant attenuation (*p* < 0.0001). Incubation with apamin and TRAM-34 did not influence dilator responses. Co-incubation of vessels with L-NNA, indomethacin, apamin and TRAM-34 resulted in attenuated dilation (*p* < 0.0001). Incubation with dorsomorphin resulted in attenuated dilation (*p* < 0.001), whilst EX-527 had no effect. Endothelial-denudation resulted in loss of the dilator component (*p* < 0.001) (**G**; *n* = 4). Two-way ANOVA followed by Sidak multiple comparisons test. Data are presented as mean ± SEM.* *p* < 0.05. ** *p* < 0.01. **** *p* < 0.0001.

To define the dilator pathways that RV-NLCs may be stimulating to potentiate endothelial-dependent dilation, inhibition studies were carried out in the presence of RV-NLCs. Incubation with L-NNA resulted in significantly attenuated dilation (*p* < 0.0001; Figure 6A). Similarly, the co-incubation of vessels with indomethacin resulted in a significant attenuation of vascular responses (*p* < 0.0001; Figure 6B). Incubation with apamin and TRAM-34 did not influence responses, suggesting that the RV-NLCs may be acting via EDH-independent mechanisms (Figure 6C). L-NNA, apamin, TRAM-34 and indomethacin resulted in a further reduction in the dilator component (*p* < 0.0001; Figure 6D). Endothelial-independent responses were unaffected. Co-incubation with dorsomorphin (AMPK inhibitor) led to a significant reduction in dilation (*p* < 0.001), resulting in a dilator capacity similar to that observed following acute pressure elevation, minus intervention (Figure 6E). In contrast, incubation with EX-527 (SIRT-1 inhibitor) did not influence responses (Figure 6F). Denudation resulted in the complete abolishment of responses, confirming that RV-NLCs potentiate dilation via endothelial-dependent mechanisms (*p* < 0.0001; Figure 6G).

## 4. Discussion

We demonstrate for the first time that RV delivered via NLCs (composed of the lipids trimyristin and triolein for improved stability and drug loading) can restore attenuated endothelial-dependent vasodilator responses of isolated small coronary vessels, following acute pressure elevation, over a longer time duration than RV solution alone. Using our ex vivo model of hypertension in conjunction with inhibition studies, we demonstrate that RV-NLCs promote the activation of both NO and COX vasodilator pathways, mediated via adenosine monophosphate-activated protein kinase (AMPK).

The production of NLCs via probe sonication is facile and easy to implement and results in uniform particles of less than 100 nm. Additionally, the process is water based and does not involve the use of organic solvents, which may lead to heightened toxicity. The primary components used were triglycerides and phospholipids, recognized for their high biocompatibility and tolerance for use within pharmaceutical applications. RV showed high drug entrapment within the NLCs as quantified by HPLC, and its presence was further confirmed with UV–VIS, fluorescence and NMR spectroscopy. The zeta potential of −25.6 signifies good stability of RV-NLCs due to electric repulsion. Our previous report has demonstrated good shelf-life stability of RV-NLCs [27]. In the presence of physiological media PSS, NLCs showed no disintegration or aggregation with zeta potential in the range of −9 mV due to the presence of an ionic layer on the surface of NLCs. Lipid-based nanoparticles are known to display thermal transitions that are compatible with physiological environments [39]. Exposure to stable RV-loaded NLCs had no overall effect on endothelial cell viability, while the incorporation of the dye within the RV-NLCs only affected viability at the highest concentrations, as previously described [40]. Previous studies have demonstrated the dose-dependent effects of RV solution on EC proliferation and apoptosis, with reports of concentrations above 50 µM resulting in 20–50% cell death following 24 h incubation [41]. We show improved biocompatibility of our RV-NLCs in comparison to RV alone, which may be attributable to loading within the NLCs, resulting in a slower, sustained release of RV over time. Uptake into HCAECs was confirmed within 30 min of exposure to the RV-NLCs, observed throughout the cytoplasm in the form of clusters. Indeed, the negatively charged surface reduces nonspecific charge–charge interplay between NLCs and the cell surface, thereby enhancing lipophilic affinity-based interaction with the cell surface, thus facilitating interaction with lipid-rich cell domains [42]. NLCs are known to be taken up by caveolae, which are abundantly expressed on endothelial cells [43]. Furthermore, the presence of triolein within the NLCs would have contributed to the modulation of the cell membrane for boosted cellular uptake 26. RV-NLCs were notably absent within the nucleus. 

In HCAECs exposed to H_2_O_2_, we demonstrated that the generation of mitochondrial and cytosolic superoxide was significantly reduced in the presence of RV-NLCs. Previous studies have observed elevated intracellular superoxide generation following brief periods of exposure (<10 min) to H_2_O_2_, potentially mediated via the NADPH oxidase and xanthine oxidase pathways, as well as through NOS uncoupling [30,44]. Our findings are consistent with previously observed results, with RV resulting in attenuated mitochondrial superoxide in HCAECs following hyperglycemia-induced mitochondrial ROS generation [45]. RV can directly remove free radicals due to the presence of hydroxyl groups in both phenyl rings of the stilbene scaffold, promoting hydrogen atom transfer (HAT) from hydroxyl groups to surrounding reactive oxidants, a common feature amongst phenolic compounds [46,47]. RV can also upregulate several molecular targets involved in the prorogation of dilation and the regulation of basal antioxidant activity, including eNOS, superoxide dismutase, catalase and nuclear factor erythroid 2-related factor 2 (Nrf2) [48]. Mitochondrial and cytosolic superoxide is a significant contributor to NO quenching and has previously been found to be increased in isolated arteries following elevated pressure, interfering with flow-mediated dilation and endothelial-dependent dilation in multiple vascular beds [49,50].

In isolated coronary arteries, following acute pressure elevation, we demonstrated a significant reduction in endothelial-dependent dilation due to increased superoxide generation, mediated via NADPH oxidase, as previously described [51,52]. Vessels were incubated with SOD or apocynin following pressure elevation to assess the contribution of superoxide and NADPH oxidase. Consistent with the previous literature, SOD incubation following pressure elevation led to the restoration of dilation [53,54]. Co-incubation with RV-NLCs resulted in the restoration of dilation to a similar magnitude as that following SOD treatment. RV-NLC incubation did not influence endothelial-independent responses. To assess any potential improvements provided by NLC encapsulation, the non-encapsulated RV solution was used for comparison. Although RV solution resulted in a larger magnitude in dilation, likely attributable to superior diffusion kinetics over time in comparison to RV-NLCs, the sustained responses were significantly less than those induced by RV-NLCs, with a nearly five-fold improvement in potency shown by the RV-NLCs (as shown by the LogEC_50_ data). Similarly, we also show that incubation in RV-NLCs can provide sustained endothelial-dependent dilator responses over longer time periods (4 h) when compared to RV solution, using isolated aortic rings from Wistar rats, after acute tension elevation, ex vivo (Supplementary Figure S5A,B), with a 300-fold increase in potency (LogEC_50_ = 1.56 × 10^−7^ M vs. 4.57 × 10^−5^ M, respectively). Endothelial-independent dilator responses (SNP, 100 µM) were maintained and unaffected by incubation in RV-NLCs. We suggest that RV loading within the NLC protected the RV, which was slowly released over a longer duration. The reduced potency of RV solution in comparison to RV-NLCs may be partly attributable to RV’s degradation. This is supported by our previous observation that RV stability is significantly reduced one hour after circulation in the ex vivo organ bath setup [15]. This degradation may partly be due to RV’s high sensitivity to photocatalytic degradation and the accumulation of several breakdown products resulting in cellular toxicity [55].

Both NO and EDH play an integral role in regulating dilation in the coronary arteries, with potential crosstalk and dynamic compensatory mechanisms between pathways [56,57,58]. It has been shown that hypertension has the potential to disrupt both NO and EDH pathways [59]. Our inhibition studies demonstrate that, following acute pressure elevation, NO is the major dilator component in coronary arteries with modulation by the COX and EDH pathways, as previously documented in vessels from aged mice [60]. Inhibition using L-NNA significantly compromised dilation, suggesting that a basal release of NO from the endothelium following acute pressure elevation has a significant dilator influence on the coronary vasculature [38]. Incubation with apamin and TRAM-34 resulted in the potentiation of dilation, a possible compensatory mechanism facilitated by NO. Indomethacin resulted in significantly improved dilation, likely attributable to its inhibitory effect on downstream COX constrictors, such as prostaglandin H_2_ and thromboxane A_2_. Following incubation in RV-NLCs (after acute pressure elevation), inhibition studies suggest that RV-NLCs can potentiate dilation via NO and COX pathways. After incubation with all four inhibitors, a small amount of dilation was still observed following pressure elevation ± treatment. This may be attributable to stimulation of the Ca^2+^ permeable cation channels, such as transient receptor potential vanilloid 4 (TRPV4), which is highly expressed in vascular endothelial cells [61]. Co-incubation with dorsomorphin resulted in a partial reduction in dilation, suggesting a partial involvement of AMPK in mediating the effects of RV-NLCs. In contrast, co-incubation with the SIRT-1 inhibitor EX-527 did not influence dilator responses, suggesting the absent participation of SIRT-1 in RV-mediated dilation. These data contrast previous findings that have attributed the effects of RV to SIRT-1; however, in vitro studies in which the effects of SIRT-1 have been observed in the coronary artery are often performed following several days/weeks of exposure to agonistic stimulus and/or treatment with RV, which is likely to modulate SIRT-1/AMPK activity. There is also considerable variability in the experimental concentrations used, with AMPK activation being found to be cell-type and concentration dependent [62]. Furthermore, whilst many reports have documented the effects of RV and its modulatory effects on SIRT-1, little is known about its molecular mechanism of action. The effects of RV are known to be receptor mediated, with several potential candidates identified to date (e.g., androgen-, estrogen- and troponin-c-receptor) [63,64,65]; in contrast, RV-NLCs are capable of being endocytosed into cells, releasing RV directly into the cytoplasmic compartment [66,67]. Activation of these cell surface receptors inherently implicates multiple signaling pathways, the mechanisms of which may express preferential aversion or affinity toward downstream targets (e.g., SIRT-1 vs. AMPK).

## 5. Conclusions

This study aimed to assess the potential of RV, loaded within trimyristin–triolein NLCs, to restore vasodilator responses in an ex vivo model of acute hypertension. Using chemico-analytical techniques, we demonstrate the synthesis of stable NLCs, with the ability to entrap and modulate RV release, as a model organic molecule. We demonstrate the antioxidant capacity of RV-NLCs, reducing both cytosolic and mitochondrial superoxide anion levels in HCAECs in vitro. Using our ex vivo model of acute hypertension, we demonstrate that RV-NLCs can restore dilation to levels comparable to those observed after SOD treatment. Furthermore, these responses were sustained over a more prolonged period in comparison to RV solution alone. Through inhibition studies, we show that RV-NLCs restore the ROS-mediated attenuation of endothelial-dependent dilator responses in a hypertensive environment via potentiation of the NO and COX pathways, mediated by AMPK. Our findings have important implications for the future design and implementation of antihypertensive treatment strategies.

## 6. Novelty and Significance

The findings of our study demonstrate the potential of novel trimyristin–triolein NLCs to entrap and release RV over sustained periods of time, within an oxidative environment in vitro, reducing both cytosolic and mitochondrial superoxide anion levels. Our data are consistent with previous observations, whereby RV solution reduced mitochondrial superoxide in HCAECs following hyperglycemia-induced mitochondrial ROS generation, highlighting the potential of RV-NLCs as an antioxidant in future therapeutic applications. Whilst the effects of RV on endothelial viability and oxidative capacity have been previously documented, our study is the first to document the effects of novel NLCs composed of trimyristin–triolein (which incurs enhanced stability, RV entrapment and cellular uptake) on HCAECs and isolated coronary arteries. Furthermore, using an ex vivo model of acute hypertension, the RV loaded within NLCs demonstrated the potential to restore and potentiate attenuated dilator responses in the coronary artery via the modulation of NO and COX pathways, partially mediated by AMPK as opposed to the conventionally accepted SIRT-1 pathway. Differences in the mode of delivery and the uptake of RV may reflect different regulatory pathways, which have implications on treatment efficacy and outcome, hence impacting future design and the implementation of antihypertensive treatment strategies.

## Data Availability

The data presented in this study are available on request from the corresponding authors.

## Supplementary Materials

The following are available online at https://www.mdpi.com/article/10.3390/biomedicines9121852/s1, Figure S1: The effects of hydrogen peroxide (H_2_O_2_) on human coronary artery endothelial cell (HCAEC) viability. Figure S2: The dose-response effect of 5-HT (10^−10^ M − 10^−3^ M), assessed in coronary arteries under normotensive pressure (60mmHg) ex vivo. Figure S3. RV-NLC stability and release profile. Figure S4. The effect of resveratrol (RV) loading on efficacy. Figure S5. The sustained dilator influence of RV-NLCs on endothelium dependent responses, in aortic rings after acute tension elevation.
