# Supplementary material for: Nanostructured Lipid Carriers Deliver Resveratrol, Restoring Attenuated Dilation in Small Coronary Arteries, via the AMPK Pathway
Source: Biomedicines. 2021 Dec 7;9(12):1852. doi: 10.3390/biomedicines9121852 (PMC8699041; doi:10.3390/biomedicines9121852)
Supplement: Supplementary file 1 [file biomedicines-09-01852-s001.zip › biomedicines-1490020-supplementary.pdf]

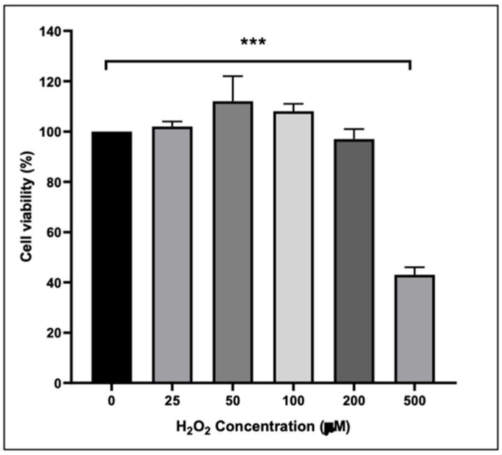

Supplementary Figure S1. Effect of H_2O_2 on oxidative reactivity in vitro. Human coronary artery endothelial cell (HCAEC) viability following exposure to hydrogen peroxide (H_2O_2; 0-500µM) for 30 min. H_2O_2 exposure had no significant effects on cell viability, other than at 500µM (P = < 0.001). Cell viability determined using Alamar blue assay (n = 3). Data normalized to control wells (untreated). One-way ANOVA followed by Bonferroni correction. Data are presented as mean ± SEM. *** P < 0.001.

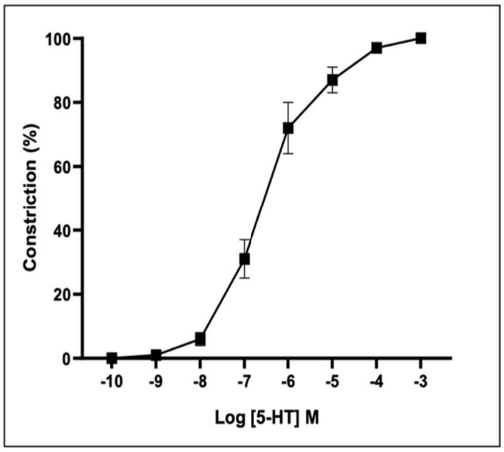

Supplementary Figure S2. Constrictor response to 5-HT in coronary arteries. The dose-response effect of serotonin (5-HT; 10^{-10} M – 10^{-3} M) in coronary arteries of young Wistar rats (2-month) under normotensive pressure (60 mmHg). All vessels constricted to 5-HT in a dose- dependent manner (n = 8). Data are presented as mean ± SEM.

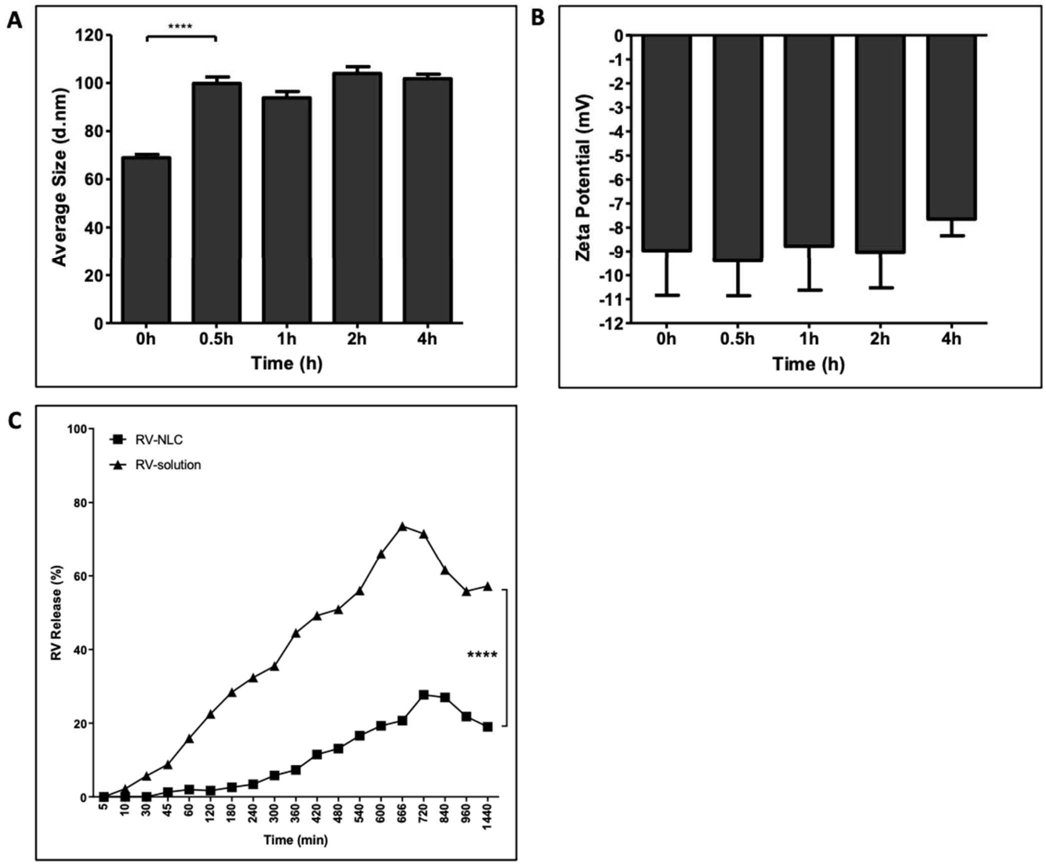

Supplementary Figure S3. Stability and electrophoretic mobility of RV-NLCs in aqueous medium. Resveratrol-loaded nano lipid carrier (RV-NLC) stability was assessed in water using dynamic light scattering and laser doppler micro-electrophoresis, pre- and post- sonication. **(A)** RV-NLC size and zeta potential were assessed in PSS and cell culture medium over 4 hours (0-, 0.5-, 1-, 2- and 4 hours). Incubation of RV-NLCs (0.45μM) in PSS led to a significant increase in size after 30-minutes (68.97 ± 1.36 nm to 98.7 ± 4.5 nm; P = < 0.0001); size was unaffected in media. **(B)** Surface charge was unaffected over the 4-hour period, indicating stability of the RV-NLCs in PSS and media. **(C)** Dissolution of RV-NLCs and RV-solution in vitro. The in vitro release profile was determined using a dialysis bag diffusion method (37 °C, pH 7.4) over 24 hours. Samples were taken at predetermined time intervals and analyzed using high- performance liquid chromatography. RV-loading within NLCs significantly reduced the drug release into the surrounding medium, with a maximum release (27.7 %) observed after 12 hours; significantly less than the maximal release observed from the RV-solution after 11 hours (71.5 %; P = < 0.0001). Data was analyzed using a two-way ANOVA. Data are presented as mean ± SEM. **** $P < 0.0001$.

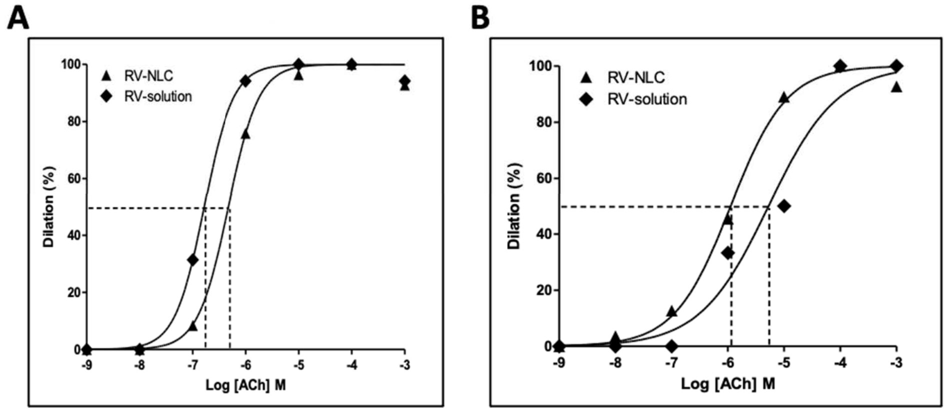

Supplementary Figure S4. Effect of RV-NLCs on initial and sustained dilator responses to ACh. **(A)** A significant shift in $LogEC_{50}$ values was observed between responses to RV-NLCs and RV- solution, with mean values of 4.73 x 10^{-7} M (0.47μM) and 1.64 x 10^{-7} M (0.16μM) respectively ($P < 0.0001$). **(B)** A significant shift in $LogEC_{50}$ values were observed between sustained responses to RV-NLCs and RV-solution, with mean values of 1.13 x 10^{-6} M (1.13μM) and 5.33 x 10^{-6} M (5.33μM), respectively ($P < 0.01$).

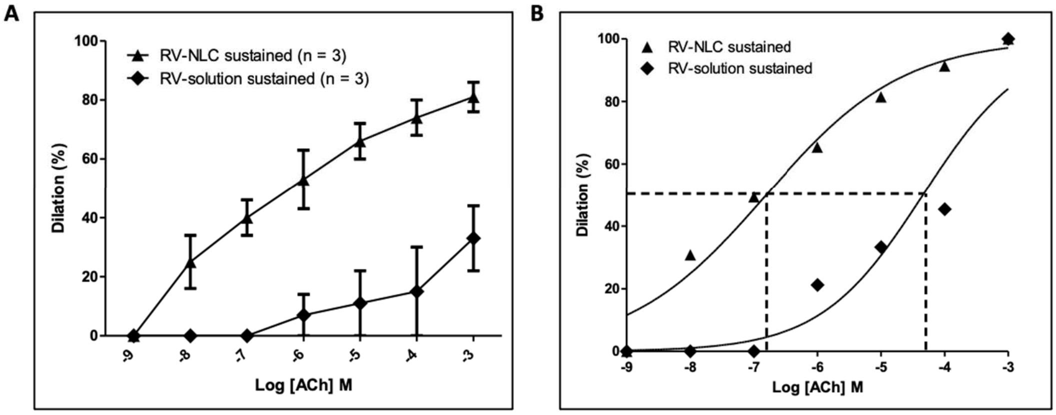

Supplementary Figure S5. Effect of RV-NLCs on sustained dilator responses of isolated aortic rings. **(A)** Incubation in RV-NLCs provide sustained dilator responses to ACh over longer time periods (4 hours) when compared to RV-solution, with a 300-fold increase in potency ($LogEC_{50}$ = 1.56 x10^{-7}M vs 4.57 x10^{-5}M, respectively) **(B).**
